# Supplementary material for: Scalable and efficient DNA sequencing analysis on different compute infrastructures aiding variant discovery
Source: NAR Genom Bioinform. 2024 Apr 25;6(2):lqae031. doi: 10.1093/nargab/lqae031 (PMC11044436; doi:10.1093/nargab/lqae031)
Supplement: lqae031_Supplemental_File [file lqae031_supplemental_file.pdf]

Appendix A Overview of quality control tools in variant calling pipelines

	Reads	Alignment	Duplicate Marking	Variants	Report
nf-core/sarek	FastQC<br>FastP	samtools stats<br>mosdepth	GATK4 Markduplicates<br>or<br>GATK4 EstimateLibraryComplexity	vcftools<br>bcftools<br>snpeff, vep	MultiQC
OvarFlow	FastQC	samtools depth<br>covariate analysis	GATK4 Markduplicates	snpeff	-
Sequana VC	-	samtools depth<br>sequana coverage	sambamba markdup	snpeff	MultiQC
TOSCA	FastQC	samtools stats<br>mosdepth<br>BamStats04<br>GATK CallableLoci	GATK4 Markduplicates	snpeff	MultiQC
OTP	FastQC	samtools flagstat<br>Sex estimation<br>GC correction<br>In-house scripts	sambamba<br>biobambam<br>or picard	In-house scripts	-

Table S1: Variant calling pipelines typically use a set of quality control tools to allow introspection of data quality and intermediate analysis steps. Here, the various tools used by the pipelines described in Table 1 are shown. All pipelines report basic mapping, duplication metrices, and variant calling metrics. nf-core/sarek, sequana VC, and TOSCA combine these into a MultiQC report.

Appendix B Table with detailed changes between Sarek 2.5.2 and 3.1.1

	Feature	Sarek 2.5.2	Sarek 3.1.1
Pre-processing	uBAM conversion	GATK4 SamToFastq	SAMtools
	FastQ splitting	Nextflow `SplitFastq()`	fastP
	UMI support	-	fgbio
	Trimming	-	fastP
	Mapping	BWA-MEM	BWA-MEM, BWA-MEM2, Dragmap
	Merging of mapped files	SAMtools	GATK4 Markduplicates(Spark), SAMtools
	Duplicate marking	GATK4 Markduplicates	GATK4 Markduplicates(Spark)
	Quality control	FastQC, SAMtools, Qualimap	FastQC, fastP, SAMtools, mosdepth, GATK4 EstimateLibraryComplexity
Variant calling	Germline	Haplotypecaller, Manta, Mpileup, Strelka2, Tiddit	CNVKit, DeepVariant, FreeBayes, Haplotypecaller (extended single-sample filtering and join-germline), Manta, Mpileup, Strelka, Tiddit
	Tumor-only	Manta, Strelka, Tiddit	FreeBayes, Manta, Mutect2, Strelka2, Tiddit
	Paired	ASCAT, ControlFreec, FreeBayes, Manta, Mutect2, Strelka2	ASCAT, CNVKit, ControlFreec, FreeBayes, Manta, MsiSensorPro, Mutect2, Strelka2, Tiddit
	Merging	Custom script with bcftools	
Annotation		VEP CADD plugin	VEP dbnsfp, Loftee, spliceAI, spliceRegion plugin
Other	Sentieon support	yes	planned for 3.3

Table S2: The updates since nf-core/sarek 2.5.2 are summarized in this table: most notably adapter trimming and UMI support were added, and read splitting was optimized and enabled for large FastQ files by using fastP. Merging of aligned BAM files across splits and lanes was incorporated into the duplicate marking process. Furthermore, individual steps allow a wider tool selection, i.e. BWA-MEM2 and Dragmap for mapping and further tools for variant calling. Sentieon support for all steps will be added with release 3.3.

Appendix C Intra-sample parallelization

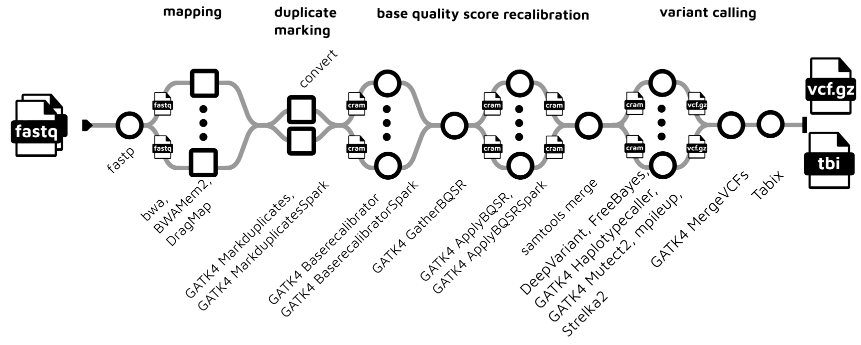

Fig. S1: The figure depicts the processing of a single sample. While Nextflow runs the analysis of each sample in parallel, intra-sample parallelization was implemented for the mapping step by splitting the FastQ files beforehand, mapping each, and merging all FastQ files belonging to one sample and duplicate marking them. Base quality score recalibration and SNP/Indel calling are run across genomic intervals, which can be either user-provided or GATK-provided interval files.

Appendix D Reducing storage requirements with CRAM

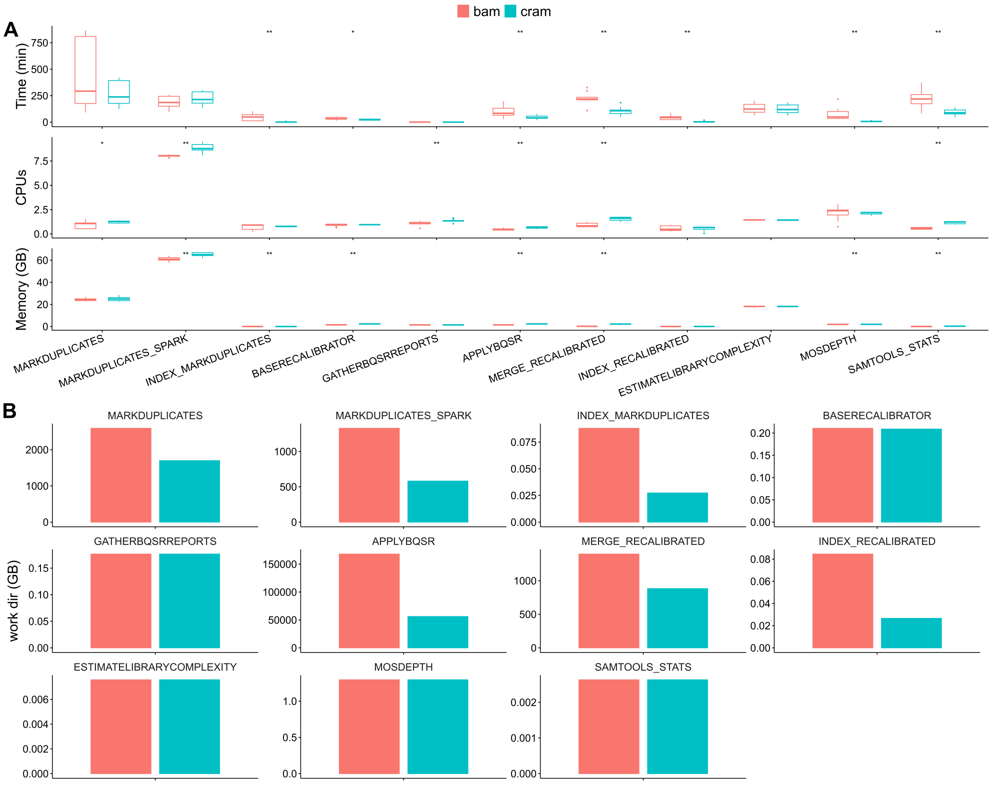

Fig. S2: Resource usage of nf-core/sarek 3.1.1 when storing intermediate data in BAM versus CRAM format for all pre-processing processes. **A)**: Average realtime, and maximum CPU and memory usage (peak_rss) as reported by the Nextflow trace file. For processes split within a sample (i.e. ApplyBQSR), the task with the highest runtime per sample is shown as the process runtime. Resource usage was compared using the paired Wilcoxon test (** $p < 0.01$, * $p < 0.05$). Seven of the eleven processes are significantly faster when using the CRAM format. Five processes have a significantly higher CPU hour usage, and three require more memory in comparison to using BAMs. **B)**: Storage was evaluated by calculating the total size of the work directories of all tasks of the respective process. The storage usage between both is identical, due to Nextflow accessing the input files via symlinks. Thus only the output is measured here for each process, which is independent of input format. Each condition was repeated three times for samples of five tumor-normal paired patients.

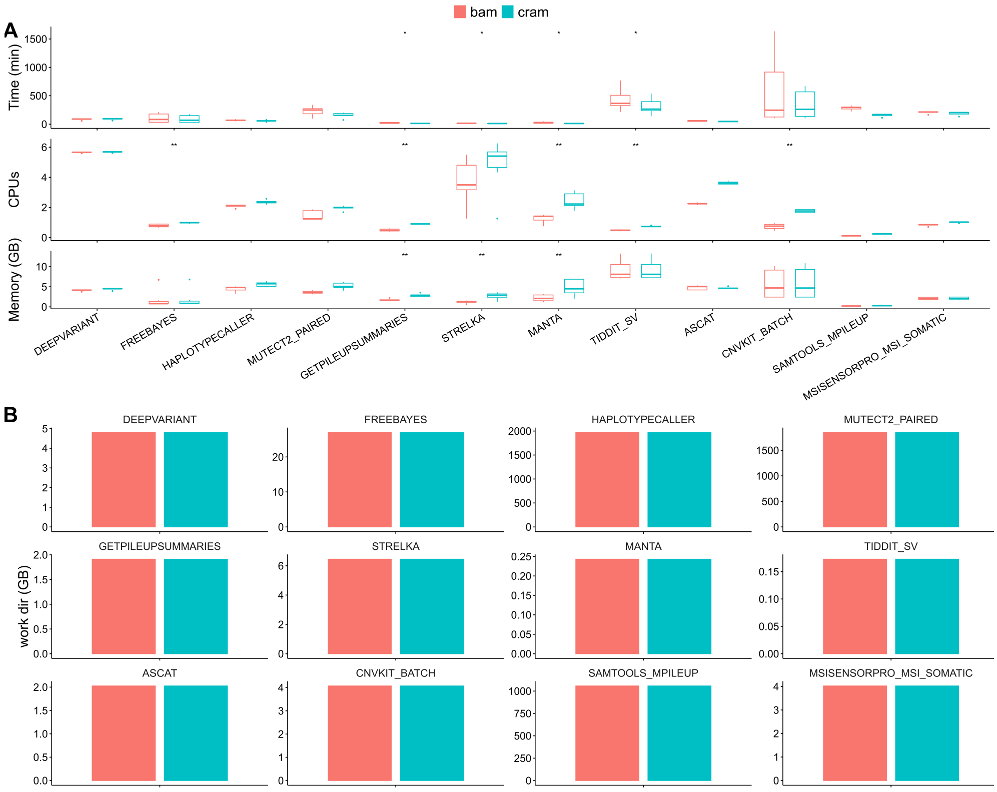

Fig. S3: Resource usage of all variant calling processes in nf-core/sarek 3.1.1 when starting from BAM and CRAM format. **A)**: Average realtime, and maximum CPU and memory usage (peak_rss) as reported by the Nextflow trace file. For processes split within a sample, the task with the highest runtime per sample is shown as the process runtime. Resource usage was compared using the paired Wilcoxon test (** $p < 0.01$, * $p < 0.05$). Four of the 12 processes are significantly faster when using the CRAM format. Six have a significantly higher CPU hour usage, and seven require more memory in comparison to using BAMs. **B)**: Storage was evaluated by calculating the total size of the work directories of all tasks of the respective process. Six processes have reduced storage requirements. Each condition was repeated three times for samples of five tumor-normal paired patients.

Appendix E Sharding FastQ files reduces runtime

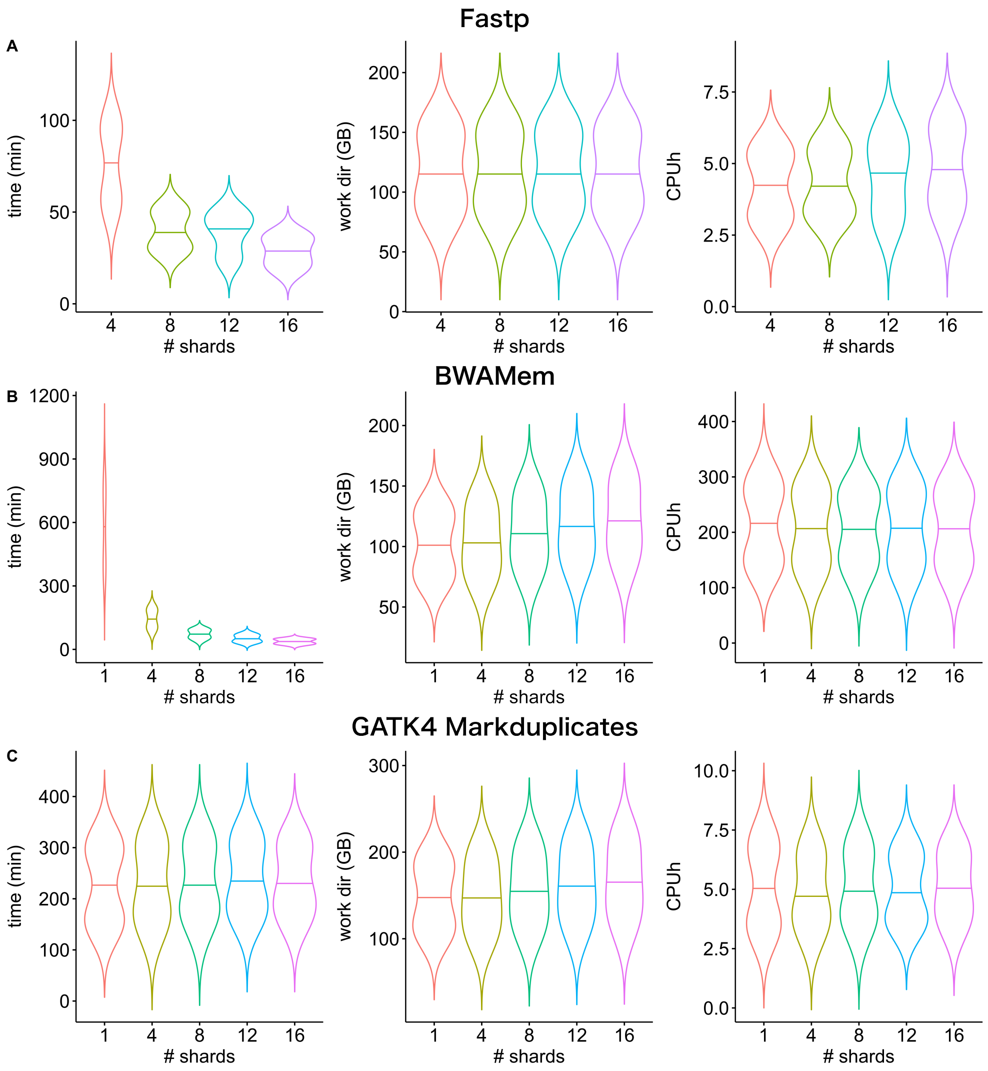

Fig. S4: Dividing the input FastQ files into increasing amounts of shards. **A)**: Resource usage of fastP during sharding of the input FastQ files. The tool was run with a different count of CPUs corresponding to the desired number of shards. **B)**: Resource usage of BWA-MEM during mapping of each shard. **C)**: Resource usage of the duplicate marking process. Merging of sharded bam files and duplicate marking is performed with GATK4 Markduplicates. CRAM conversion is done with SAMTools. The violin plots show computations on tumor-normal paired samples of five patients. The time was evaluated by summing up the highest realtime per task per sample as reported by the Nextflow trace report. The work directory size and CPU hours are the sums of all involved tasks.

Appendix F Splitting by intervals reduces runtime

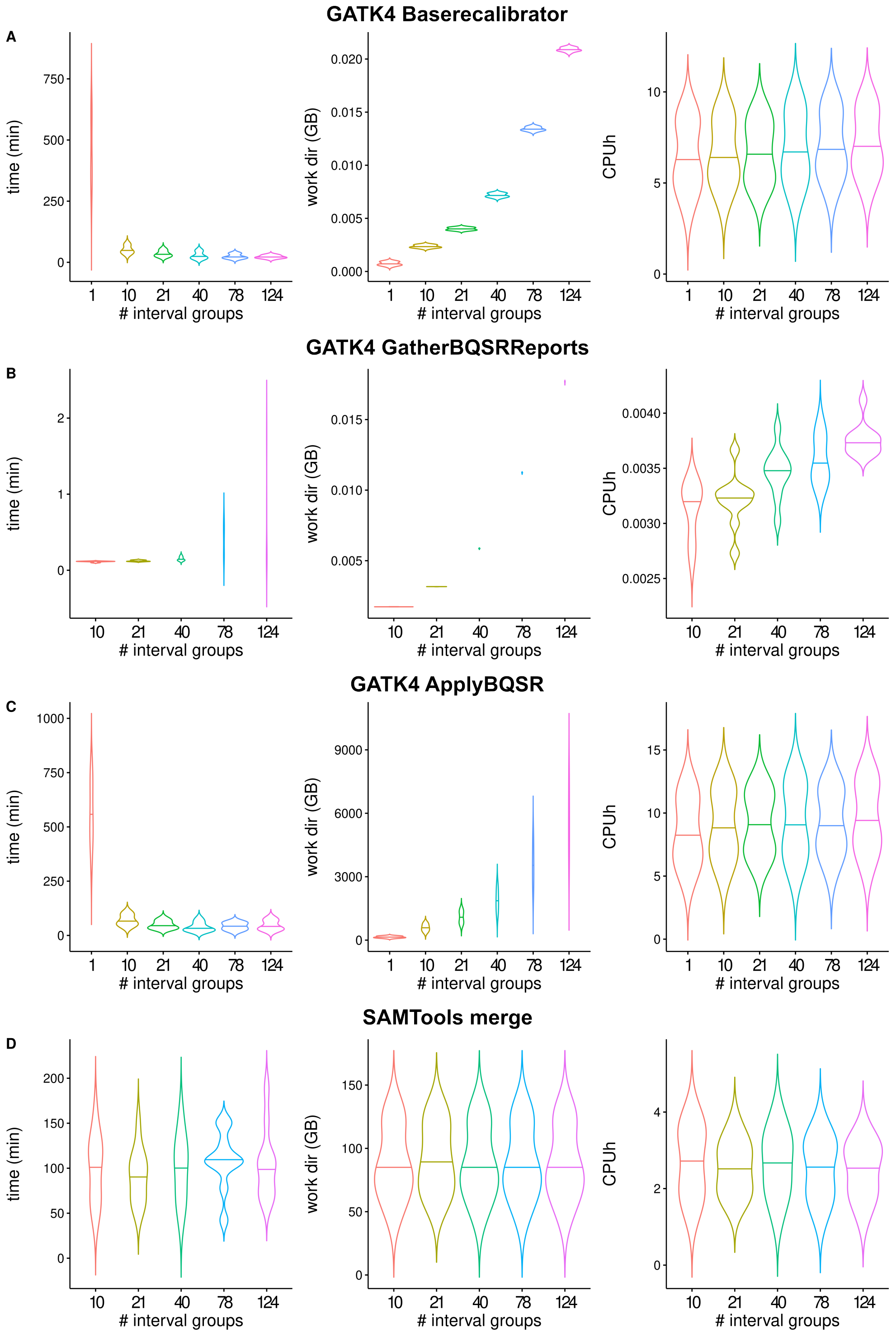

Fig. S5: Parallel processing of interval groups reduces runtime **A)**: GATK4 BaseRecalibrator runtime decreases with an increasing number of interval groups. Storage space requirements increase, while CPU hours stay consistent. **B)**: For GATK4 GatherBQSRReports all three metrics increase with an increasing number of interval groups. **C)**: For GATK4 ApplyBQSR the runtime decreases with an increasing number of interval groups. Storage space requirements increase, while CPU hours stay consistent. **D)**: For SAMTools Merge all three metrics are consistent across the number of interval groups. The violin plots show computations on tumor-normal paired samples of five patients for each tool. The time was evaluated by summing up the highest realtime per task per sample as reported by the Nextflow trace report. The work directory size and CPU hours are the sums of all involved tasks.

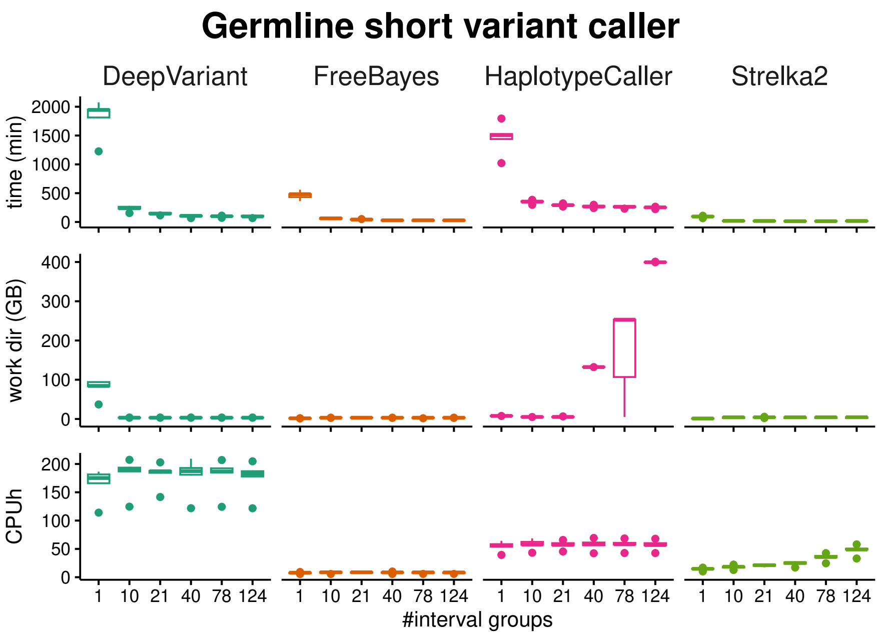

Fig. S6: Effect of parallelizing computations across interval groups on germline variant calling processes, which include the respective variant caller followed by GATK4 MergeVCFs. FreeBayes VCFs are sorted before merging. GATK4 HaplotypeCaller is followed by GATK4 CNNSCoreVariants and GATK4 FilterVariantTranches. All variant callers speed up on parallel processing across 10 interval groups. DeepVariant and HaplotypeCaller speed up further with fewer interval groups. Storage usage decreases for DeepVariant for 10 interval groups and remains stable. FreeBayes and Strelka2 have similar storage usage across parallelization. For VCFs called by HaplotypeCaller storage usage increases. CPU hours are similar across degrees of parallelization with an increase measured for Strelka2. The violin plots show computations on normal samples of five patients. The time was evaluated by summing up the highest realtime per task per sample as reported by the Nextflow trace report. The work directory size and CPU hours are the sums of all involved tasks.

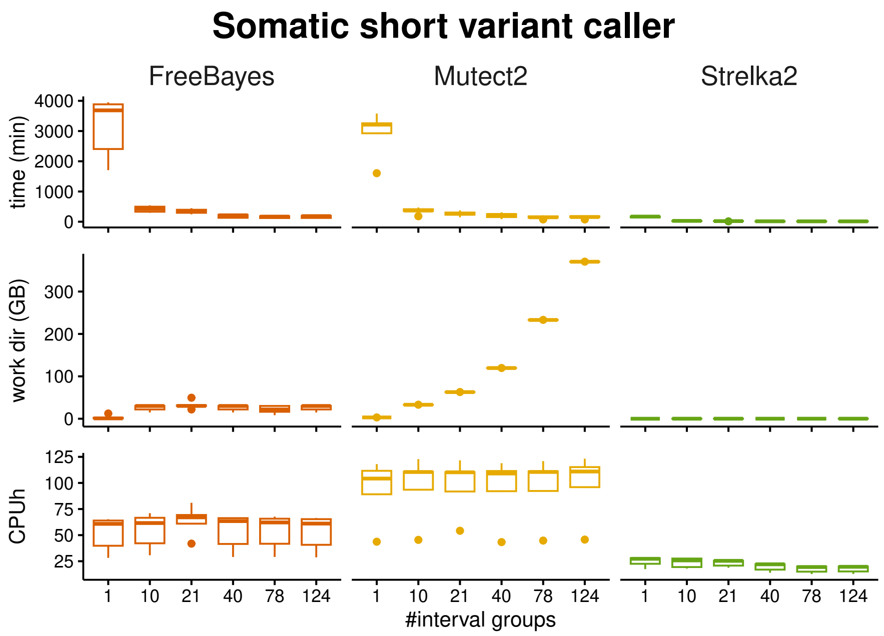

Fig. S7: Effect of parallelizing computations across interval groups on somatic variant calling processes, which include the respective variant caller followed by GATK4 MergeVCFs. FreeBayes VCFs are sorted before merging. All variant callers speed up on parallel processing across 10 interval groups. FreeBayes and Mutect2 further speed up with 21 interval groups. Storage usage increases for FreeBayes for 10 interval groups and remains stable. For Mutect2 storage usage increases with increasing interval groups. It remains stable for Strelka2. CPU hours are similar across degrees of parallelization with a decrease measured for Strelka2. The violin plots show computations on tumor-normal paired samples of five patients. The time was evaluated by summing up the highest realtime per task per sample as reported by the Nextflow trace report. The work directory size and CPU hours are the sums of all involved tasks.

Appendix G Benchmarking against truth datasets

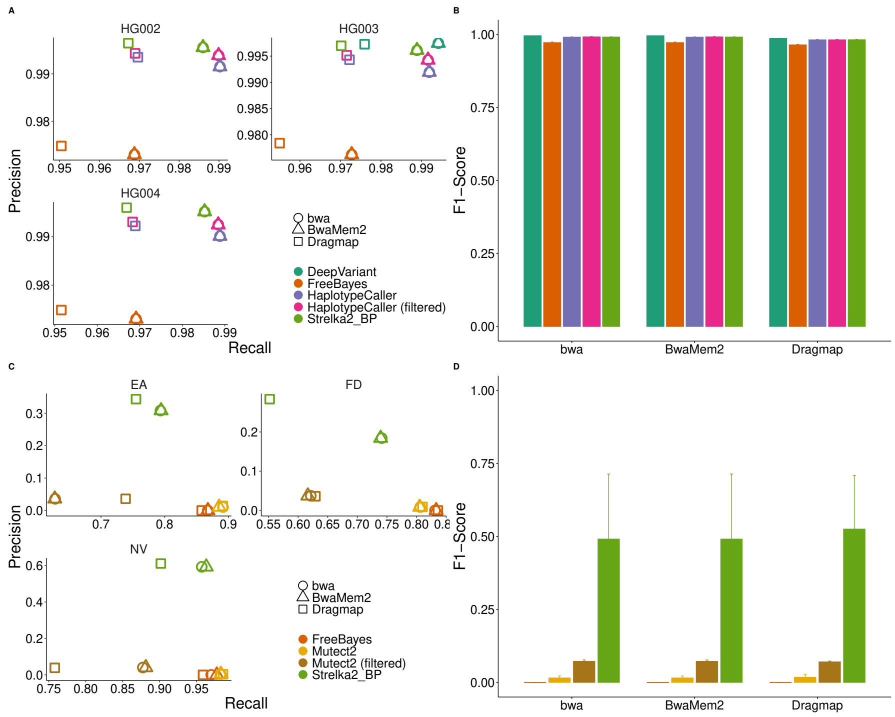

Fig. S8: Germline and somatic variant calling evaluation of high-confidence calls using ground-truth benchmarking data with respect to Indels. **A,B**: The germline track of the pipeline was evaluated using 3 WGS GiaB datasets (HG002-HG004). The average precision, recall and F1-score values across all the samples are plotted respectively. **C,D**: The paired calling track was evaluated using three tumor-normal WES pairs (EA, FD, NV) from SEQ2C.

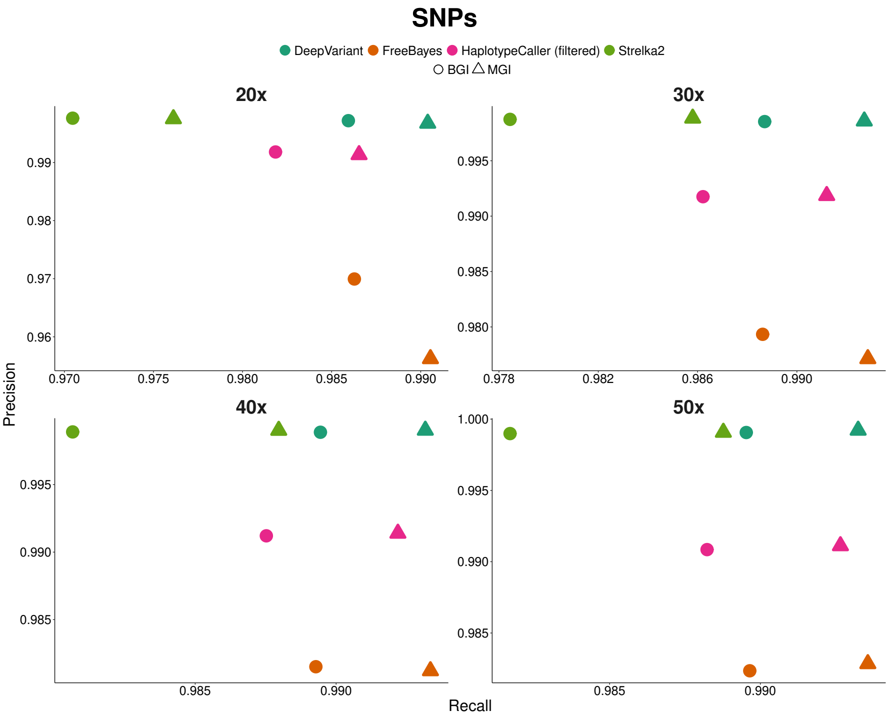

Fig. S9: Germline variant calling evaluation of high-confidence calls using ground-truth benchmarking data with respect to SNPs. Samples from MGISeq and BGISeq500 were mapped with BWA-MEM. Different coverages were used as input. For all investigated coverage values FreeBayes and DeepVariant have the highest recall. Strelka2 and DeepVariant show the highest precision values for all samples.

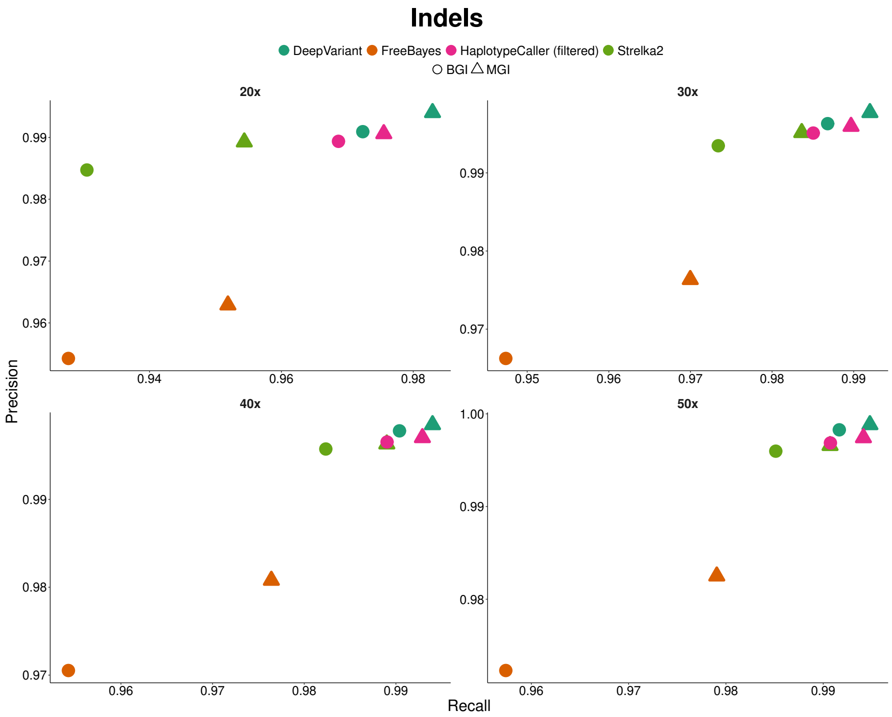

Fig. S10: Germline variant calling evaluation of high-confidence calls using ground-truth benchmarking data with respect to Indels. Samples from MGISeq and BGISeq500 were mapped with BWA-MEM. Different coverages were used as input. For all investigated coverage values HaplotypeCaller and DeepVariant have the highest recall, followed by Strelka2. MGI samples analyzed with DeepVariant had the highest precision values.

Appendix H Comparison of CNV calls against PCAWG samples

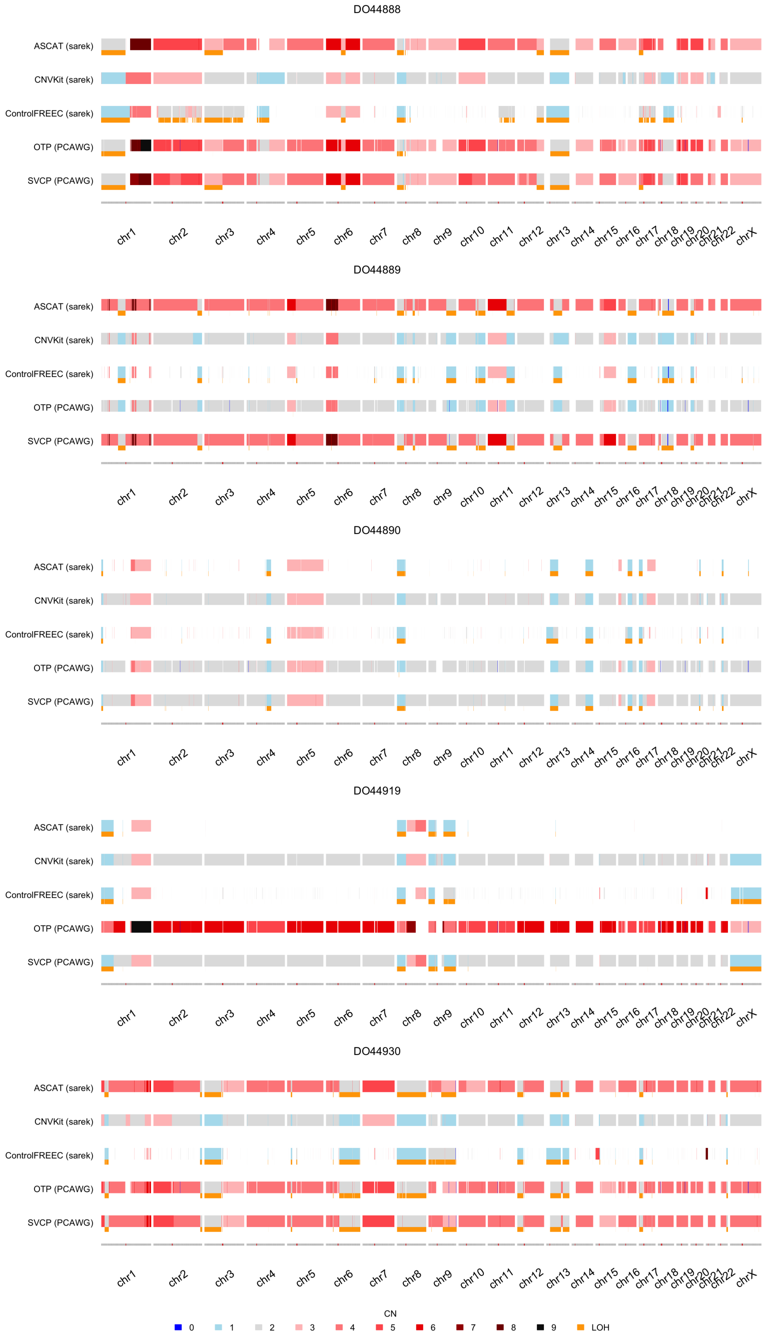

Fig. S11: Comparison of copy number calls obtained with nf-core/sarek using ASCAT, Control-FREEC, and CNVKit to the ones from the PCAWG study downloaded from the ICGC portal. For the latter, there are two results files available for each patient respectively, one called with the OTP pipeline, one called with the Sanger pipeline. The PCAWG calls agree for 3 patients. For DO44890 all calls across all pipelines and tools agree. For DO44930 and DO44888, the nf-core/sarek ASCAT calls are similar to both the OTP and Sanger pipeline based calls, the CNVKit and Control-FREEC calls differ, but are similar towards each other. For DO44889 the nf-core/sarek and Sanger pipeline calls overlap, as well as the OTP calls and nf-core/sarek Control-FREEC and CNVKit calls. Lastly, for DO44919, all nf-core/sarek calls overlap with the Sanger pipeline results, the OTP pipeline results differ.
